# Diabetes Mellitus-Related Dysfunction of the Motor System

**DOI:** 10.3390/ijms21207485

**Published:** 2020-10-11

**Authors:** Ken Muramatsu

**Affiliations:** Department of Physical Therapy, Kyorin University, 5-4-1 Shimorenjaku, Mitaka, Tokyo 181-8612, Japan; k-muramatsu@ks.kyorin-u.ac.jp

**Keywords:** diabetic neuropathy, central nervous system, motor cortex, corticospinal tract, alpha motoneuron, gamma motoneuron, rehabilitation

## Abstract

Although motor deficits in humans with diabetic neuropathy have been extensively researched, its effect on the motor system is thought to be lesser than that on the sensory system. Therefore, motor deficits are considered to be only due to sensory and muscle impairment. However, recent clinical and experimental studies have revealed that the brain and spinal cord, which are involved in the motor control of voluntary movement, are also affected by diabetes. This review focuses on the most important systems for voluntary motor control, mainly the cortico-muscular pathways, such as corticospinal tract and spinal motor neuron abnormalities. Specifically, axonal damage characterized by the proximodistal phenotype occurs in the corticospinal tract and motor neurons with long axons, and the transmission of motor commands from the brain to the muscles is impaired. These findings provide a new perspective to explain motor deficits in humans with diabetes. Finally, pharmacological and non-pharmacological treatment strategies for these disorders are presented.

## 1. Introduction

**Figure 1:**
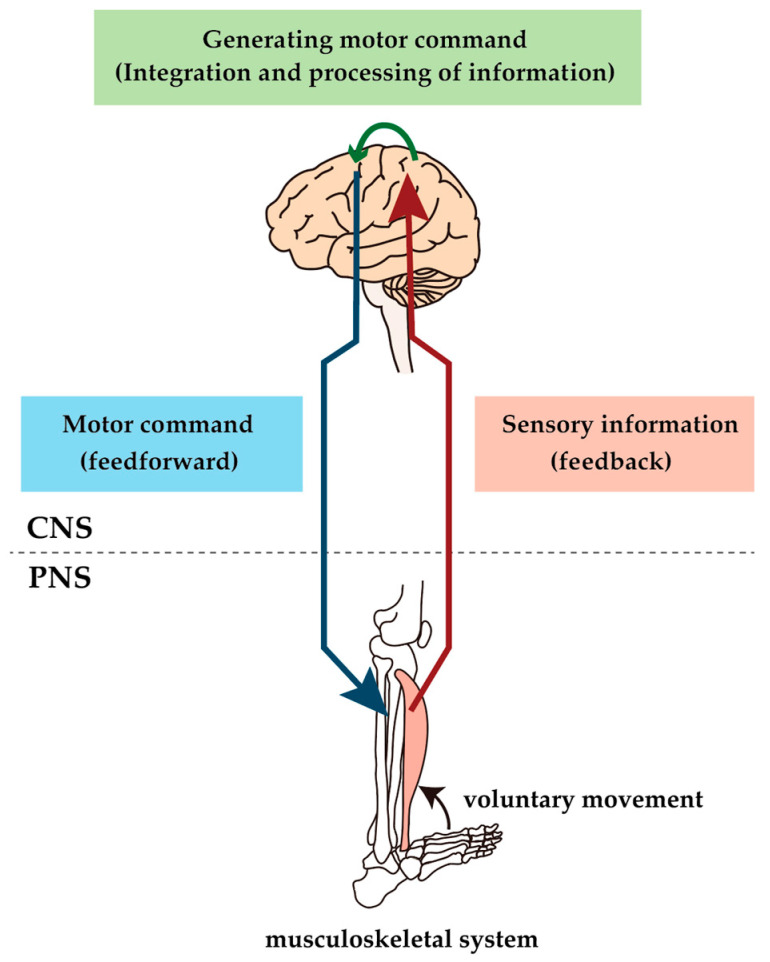
Conceptual diagram of sensorimotor control. Voluntary movements are produced by sending motor commands created by the brain through the spinal cord via motor descending pathways to the muscles, which result in muscle contraction. The result of the movement is feedback to the brain via the peripheral sensory nerve and ascending pathway, and the brain uses this information to make appropriate modifications to the motor commands. The constant activity of this loop causes voluntary movement to be performed; lesions at any level of the loop would impair voluntary movement.

Diabetic neuropathy (DN) affects both somatic and autonomic nerves in a distal, symmetrical form that progresses following a fiber-length-dependent pattern. It is one of the major complications in humans with diabetes [1]. Humans with diabetes, especially those with DN, show several motor dysfunctions, such as an increased risk of falling, altered gait and balance, and increased body sway [2,3,4]. Diabetes is also associated with a significant increase in the risk of physical disability [5,6]. Since control of movement is achieved through sensory-motor control brought about by the interaction between the peripheral (PNS) and the central nervous systems (CNS), such motor deficits can appear wherever the PNS and the CNS are affected (Figure 1). However, much attention has been paid to the causes of motor impairment in humans with diabetes in relation to sensory neuropathy. In contrast, motor nervous system involvement has received little attention. Similarly, more attention has been paid to abnormalities in the PNS than in the CNS, which may be due to several factors. First, symptoms of DN occur in the distal part of the extremities, where sensory deficits predominate and the primary clinical problems are almost exclusively centered on neuropathic pain and loss of sensation [7]. Conversely, population-based studies have reported clinically evident motor dysfunction, including the inability to stand on heels, which reflects muscle weakness of the ankle dorsal flexors in only 1–6% of humans with diabetes [8]. Therefore, motor symptoms are believed to be a rare manifestation associated with severe DN. Second, experimental studies have also shown data supporting the hypothesis that the motor nervous system is more resistant to DN than the sensory nervous system [9,10]. These differences may be due to the anatomical difference between motor and sensory neurons. The cell body of dorsal root ganglion (DRG) neurons are located outside of the blood–brain barrier (BBB), whereas motor neurons (MNs) are located within the ventral horn of the spinal cord and inside the BBB, which keeps it away from systemic metabolic and oxidative stressors [11]. In other words, the systems that control voluntary movement within the CNS are thought to be largely unaffected by diabetes. Therefore, if the relationship between the CNS and motor deficits in humans with diabetes has ever been mentioned, it has only been discussed in the context of the association of diabetes with stroke. Diabetes increases the risk and severity of stroke and causes delayed functional recovery from stroke [12,13,14]. Any considerations of a direct impact of DN on the CNS have not been adequately researched.

However, recent advances in research seem to be changing this situation. The first is the finding of muscle weakness in the lower extremities of humans with DN. Rather than relying on behavioral analysis, quantitative analysis using isokinetic dynamometers revealed weakness of both, the ankle dorsal and plantar flexor muscles and of the knee extensors and flexor muscles in humans with type 1 or type 2 diabetes [15,16]. Muscle weakness in humans with diabetes is thought to be caused by a combination of DN and disorders that occur in the muscle itself, such as an increase in intramuscular fat deposits [17]. Second, as research on cognitive impairment in humans with diabetes and its relationship to Alzheimer’s disease has progressed, it has become clear that diabetes also affects neurons and glial cells of the CNS, resulting in dysfunction and cell death. These mechanisms have mainly been investigated in hippocampal neurons [18,19,20,21,22,23]. In addition, recent advances in neuroimaging, such as diffusion tensor imaging, have revealed that structural changes include not only areas associated with cognitive function, but also the motor and sensory cortices, basal ganglia, cerebellum, brainstem, and spinal cord, which are essential in generating and transmitting motor commands [24,25,26,27]. Furthermore, more direct data reveal changes in motor representations in the motor cortex and impaired conduction of the corticospinal tract (CST) in rodents with type 1 diabetes induced by streptozotocin (STZ) administration [28,29].

Given this context, CNS involvement cannot be further ignored when trying to understand the mechanisms of diabetes-related movement disorders. In particular, there is a need to know the motor commands generated by the interaction between several brain regions and their modulation as well as the effects of diabetes on the flow of motor commands from the brain to the spinal cord and from the spinal cord to the muscles; both elements play an essential role in voluntary movement [30,31]. For the former, there is a recent and valuable review that comprehensively summarizes changes in various parts of the brain related to motor control in humans with diabetes and models of diabetes [32]. However, there is no review summarizing the alteration of motor pathways associated with diabetes. Therefore, this review addresses the effects of diabetes on the cortico-muscular pathway, which includes the primary motor cortex (M1); the CST, which conducts motor commands generated in the brain directly to the spinal cord; and MNs, which transmit these commands from the spinal cord to skeletal muscles [31,33,34]. This review also discusses the relationship between the cortico-muscular pathways and movement disorders and potential treatment strategies. In addition, since studies on motor system disorders associated with diabetes have been best investigated in animal models, data from experimental models have been added. Characteristics of the experimental models discussed in the review are summarized in Table 1.

## 2. Alteration of the Neuromuscular Pathway

MNs are neuronal cells located in the brainstem and ventral horn of the spinal cord that control skeletal muscles and a variety of motor behaviors (this review only deals with spinal MNs; spinal MNs are referred to as MNs). MNs have a long axon that extends peripherally and their activity directly leads to muscle contraction via the release of acetylcholine (ACh) at the neuromuscular junction (NMJ) [34]. Following ACh release, the action potential that spreads along the muscle cell plasma membrane and intracellularly into the transverse tubule system initiates the release of Ca^2+^ from the myoplasmic reticulum. The calcium signals activate the contraction–relaxation cycle of muscle fibers. This sequence of processes is known as “excitation-contraction coupling” [36]. There is no alternate route other than MNs to conduct commands from the CNS to the muscles in the periphery. Therefore, motor behaviors have to be expressed via the activation of MNs, referred to as the “final common path” of the CNS [37]. Therefore, if MNs are damaged and the transmission of motor commands is disrupted, the muscle fibers they innervate will experience motor paralysis.

**Figure 2:**
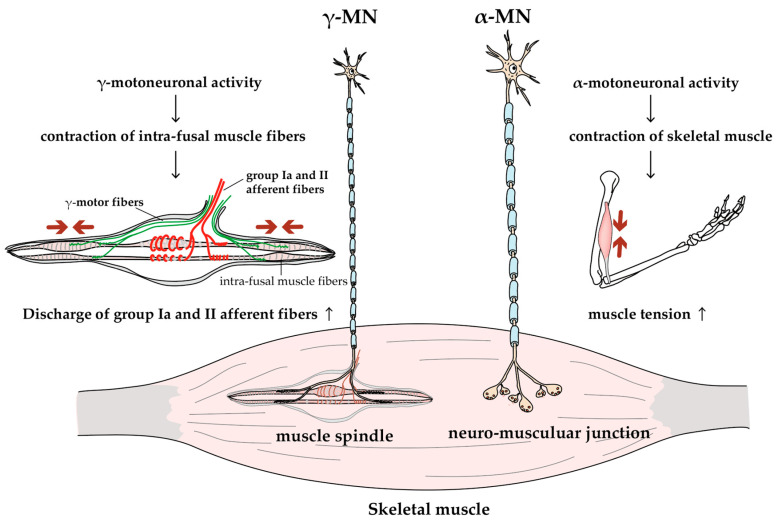
Function of alpha and gamma MNs. MNs can be divided into two groups: larger α-MNs and smaller γ-MNs. α-MNs innervate skeletal muscles, and their activity generates muscle tension and joint movements; γ-MNs innervate intrafusal fibers of muscle spindles, and their activity increases the group Ia and II afferent discharges (i.e., increases the sensitivity of muscle spindles [38]).

MNs are classified into α- and γ-MNs based on which cells they target (Figure 2). The α-MNs innervate extrafusal muscle fibers, which generate tension by contracting to generate skeletal movement. A single α-MN drives a subset of muscle fibers within a muscle, thereby defining the concept of the motor unit. It has been established that all muscle fibers of a motor unit exhibit a unique histochemical profile. Motor units are divided into three types based on their physiological type and histochemical profile: type S (slow-contracting and fatigue-resistant) motor units have type I muscle fibers, type FR (fast-contracting and fatigue-resistant) motor units have type IIA muscle fibers, and type FF (fast-contracting and fatigable) motor units have type IIB muscle fibers [38]. The CNS controls muscle strength by modulating the firing frequency and the number of recruited motor units. Meanwhile, γ-MNs innervate the intrafusal muscle fiber of the muscle spindle, which acts as a sensor for the amount and rate of change in muscle length. The activities of γ-MNs maintain and modulate the sensitivity of muscle spindles [39]. 

**Figure 3:**
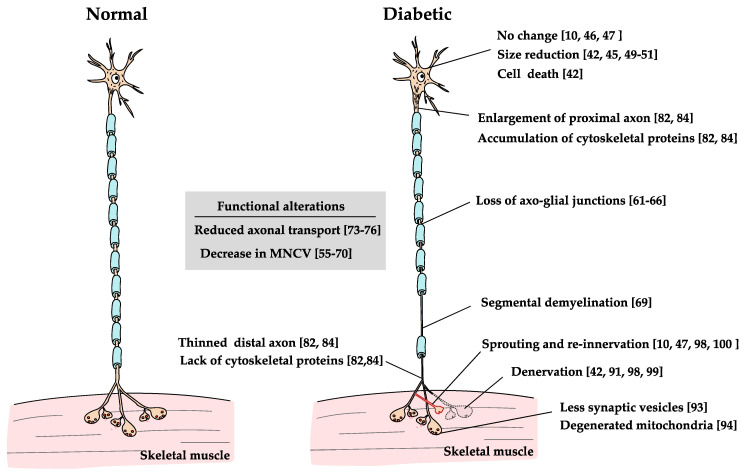
Effects of diabetes on MNs. The effects of diabetes on MNs lead to changes in various parts of the neurons [10,42,44,45,46,48,49,50,54,55,56,57,58,59,60,61,62,63,64,65,66,67,68,69,72,73,74,75,81,83,90,92,93,97,98,99]. The changes are axon length-dependent neuropathy, which is similar in many ways with DRG neurons.

Due to the anatomical features of MNs, their cell bodies belong to the CNS and their axons and nerve endings, to the PNS. Therefore, their CNS and PNS parts are probably affected differently. Motor nerve fibers and their terminals belonging to the PNS may be injured by similar mechanisms to those that affect the sensory nervous system. In sensory neurons, the pathogenesis of DN-associated cellular damage is linked with various cascades activated in response to hyperglycemia, such as polyol pathway hyperactivity, reactive oxygen species (ROS), and advanced glycation end-products (AGEs). Additionally, disorders of insulin signaling and proinflammatory processes are related to the cause and progression of DN [40]. Though multiple factors are in play, all ultimately produce oxidative stress and inflammation in cells, which is believed to be the main cause of cellular damage [41]. Effects of diabetes on MNs discussed below are summarized in Figure 3.

### 2.1. Cell Bodies of MNs

The effects of diabetes on the cell bodies of MNs vary considerably from report to report and remain controversial. In an autopsy study, humans with long-term diabetes did not only show a predominant degeneration of peripheral axons, but also degeneration of MNs, where the remaining neurons had swollen or displaced nuclei and cytoplasmic fat- and Periodic Acid-Schiff-positive deposits [42]. In contrast, in an experimental study on 8- to 9-month-old mice with STZ-induced diabetes, the size and number of cell bodies were unchanged on a long-term basis [10]. Furthermore, the size reduction of cell bodies of MNs and motoneuronal loss was not observed in this study; however, the authors reported an increased expression of molecules such as heat shock protein 27, Receptor for AGEs (RAGE), and Poly (ADP-ribose) polymerase, which are often found in degenerated neurons [10]. Compared to the same group’s study that examined sensory neurons in the same diabetic model, the present study found that motoneuronal injury is apparently minor when compared to the severe degeneration of sensory neurons [7,9]. In addition, intracellular recording from α-MNs in type II diabetic Zucker rats indicates that most of the electrophysiological properties of α-MNs remain mostly intact [43]. Thus, at least in animal models of diabetes, neurological damage does not appear to be severe enough to result in neuronal loss. There may be changes that do not lead to cell loss, such as cell atrophy, but data remain conflicting. A study reported that only 4 weeks of STZ-induced diabetes in rats resulted in reduced perikaryal volume of MNs of the fifth lumbar segment [44]. Another study, however, indicated that 4 weeks of STZ-induced diabetes did not reduce the cross-sectional area of lower lumbar MNs other than that of the bulbocavernosus MNs, which were associated with low levels of testosterone and were prevented by the administration of insulin [45]. Finally, as mentioned previously, other studies have reported that the size of cell bodies remains unchanged in long-term, 8- to 9-month STZ-treated mice [10,46]. These studies, however, did not examine isolated motor nuclei innervating specific muscles, instead pooling different motor nuclei together. Because of the complex localization of MNs in the ventral horn of rodents, there is a strong possibility that this sample included MNs with resistance to DN [47]. Conversely, cell body atrophy has been reported in our recent studies that have used retrograde tracers to selectively analyze the MNs that innervate muscles at the end of the limb, which are the most likely targets of diabetes [48,49,50]. However, since these studies exposed tracers to severed axons, cell bodies may have been atrophied due to the effects of axon disconnection [51]. 

Thus, effects of diabetes on cell bodies of MNs vary depending on the subject of the study and the method used. In addition to experimental methods, various factors, such as severity and duration of diabetes, hypoglycemia, and differences in animal species, may also play a role. For example, BB rats with hypoglycemia are known to have selective severe motor neuropathy and MN loss; therefore, complications associated with diabetes treatment may make it difficult to interpret MN disorders [52,53]. The mechanism by which MNs may be less susceptible than sensory neurons to the effects of diabetes is not known. So far, differences in their anatomical location, including their relation to the BBB, and differences in protection and nutrition of myelinated axons by Schwann cells are suspected to be involved [11].

### 2.2. Motor Axon 

The most commonly reported motoneuronal dysfunction in humans with diabetes is decreased motor nerve conduction velocity (MNCV) [54]. Since peripheral nerve conduction velocity is reduced despite the absence of symptoms of neuropathy, MNCV is widely used as the most sensitive indicator of DN [55,56]. Similar to sensory neurons, MNCV abnormalities in humans with DN are diffuse over the total length of the nerve, but more intense in distal segments [57]. The mechanism underlying the decrease in MNCV remains unclear as it is one of the earliest dysfunctions without morphological changes leading to demyelination and axonal degeneration. Similar to humans with diabetes, the functional and morphological changes that occur in the MN are observed from the earliest stages of MN neuropathy in diabetic models. The earliest changes in MN appearance are reduced blood flow to nerve fibers. As early as three days after causing STZ-induced diabetes, endoneural blood flow is reduced. Furthermore, after 1 week of diabetes, ACh-induced vasodilation has been found to be impaired [58]. Subsequently, a decrease in nerve conduction velocity occurs in as little as two to four weeks [58]. It is likely that the early decrease in MNCV may be caused by a nodal dysfunction of motor fibers, including axonal swelling and nodal bulging of the axon. The latter has been associated with an increased polyol pathway, decreased activity of the Na^+^/K^+^-ATPase, and oxidative stress [58,59,60]. These early functional deficits and structural alterations are readily reversible by the administration of insulin, but in diabetic animals with more than a few months of disease, the functional and morphological abnormalities become irreversible [60]. In chronic diabetic BB rats, peripheral axons showed delamination and degeneration of the nodal myelin that induced a loss of axo-glial junctions, which comprise the paranodal voltage channel barrier separating nodal Na^+^ channels from paranodal K^+^ channels. Loss of axo-glial junctions allows lateral displacement of Na^+^ channels across the paranodal barrier into the paranodal domain where K^+^ channels are distributed, increasing with the duration of diabetes, which suggests a decreased concentration of Na^+^ channels at the nodal domain and reduced nodal Na^+^ permeability [60,61]. These structural changes in the paranodal apparatus are clearly observed in type 1 diabetic rats and humans, but less so in type 2 diabetes models. Furthermore, these were prevented by the administration of aldose reductase inhibitors, local application of insulin, and administration of proinsulin C-peptide [62,63,64,65,66]. Therefore, these changes are thought to be associated not only with hyperglycemia, but also with insulin and C-peptide signal deficiencies [66]. In addition, a variety of other causes seem to cause a decrease in MNCV, including AGE-RAGE interactions [67]. As mentioned above, the slowing of MNCV observed in early stages of DN is due to damage that occurs in the paranodal region. However, as the disease progresses, segmental demyelination and axonal atrophy occur, causing slowing of MNCV and conduction block [68]. 

Little is known on how the decline in MNCV affects motor function. One study showed that humans with type 1 diabetes had slower MNCV and lower motor unit discharge frequency than their controls. This apparently led to impaired activation of muscles and decreased endurance during isometric fatigue [69].

Another axonal disorder that cannot be ignored is reduced axonal transport. It is related with transporting materials such as proteins, mRNA, lipids, membrane-bound vesicles, and organelles that are synthesized in the cell body. It also helps maintain communication between the cell body and synaptic terminals [70]. It has been considered that these transport systems play an essential role in maintaining normal neuronal functions [70,71]. Axonal transport has been reported to be reduced in models of diabetes. For example, fast retrograde axonal transport is reduced in STZ rats after only a week, and this reduction is greater in MNs than in DRG neurons [72]. In fact, 2 -week STZ rats show a decrease in the number of MNs retrogradely labeled by cholera toxin B subunit delivered to the cell body via axonal transport [73]. The dysfunction of fast retrograde axonal transport may reduce the transport of trophic factors, such as nerve growth factor and neurotrophin-3, which may play a role in diabetes-induced damage to the neurons [74]. Since these trophic factors are known to be necessary for MN survival, a decrease in trophic factors is expected to contribute to their dysfunction [75]. At 4–6 weeks, STZ-induced diabetes shows impairment of both anterograde and retrograde axonal transport of peripheral axons [76,77,78]. In particular, the delay of anterograde slow axonal transport of neurofilament proteins, tubulin, and other proteins is considered to be associated with a change in axonal caliber [79,80,81,82]. Transport impairment results in a larger number of cytoskeletal proteins, such as neurofilaments and microtubules, to accumulate in the proximal region of the axon, causing it to increase in size. Moreover, fewer cytoskeletal proteins reaching the distal axons cause it to show a size decrease. These dysfunctions result in hypertrophy of the proximal region of the MN axons, while the distal portion is thinned out [81,83]. This is considered an important causative factor of the proximodistal phenotype of DN. Impaired axonal transport, coupled with dysfunction in Schwann cells and other cells, also affects the delay in peripheral nerve regeneration due to diabetes [84]. The pathogenesis of axonal transport dysfunction is known to involve multiple mechanisms, including protein aggregation, excitotoxicity, oxidative stress, mitochondrial dysfunction, inflammation, and defects in axonal transport [85]. In models of diabetes, glycation of axonal cytoskeletal proteins, such as tubulin, neurofilaments, and actin, stagnate axonal transport [86]. Oxidative stress also plays an important role in axonal transport dysfunction [87]. Meanwhile, slow axonal transport in STZ-induced diabetes is probably not affected by RAGE [88]. The effects of treatment on these axonal transport disorders have been investigated in relatively short-term models of diabetes. These studies have reported that retrograde transport disorders have been restored by insulin administration in 1 week STZ rats. Furthermore, anterograde axonal transport of choline acetyltransferase has been recovered by treatment with an aldose reductase inhibitor in 3-week diabetes and 6 weeks of prevention [72,89]. 

### 2.3. NMJ and Muscle Fibers

Perhaps because the NMJ is located in the most terminal part of the motor nerve fibers, it appears to be impaired from the early stages of DN. In a recent study, motor unit number estimation from foot muscles of type 1 diabetes subjects showed reduced motor unit numbers early on, even before the onset of DN symptoms [90]. These alterations have also been detected by a morphological study, which examined biopsied muscle from acute, recently-diagnosed juvenile diabetes (3–26 weeks from acute symptoms of diabetes). The study reported morphological abnormalities of NMJs and neurogenic degenerated muscles [91]. Additionally, sprouting fibers and growth cones extended around the nerve terminal [91]. Such an alteration of the NMJ is observable in experimental models of diabetes. Intramuscular axons, after only 2 weeks of STZ-induced diabetes, have shown signs of demyelination and disruption of mitochondria occurring at the axon and nerve terminal, showing fewer synaptic vesicles and degenerated mitochondria [92,93]. The release of ACh from the motor nerve terminal also decreased after 4–8 weeks of STZ-induced diabetes in mice [94]. Furthermore, both the resting membrane potential and miniature end-plate potentials were reduced in muscle fibers [93]. 

Although the functional abnormalities of the NMJ observed in the early stages of DN may not be sufficient to cause overt motor symptoms, the chronic phase of DN produces muscle denervation and degeneration, perhaps decisively affecting muscle weakness [95]. In particular, lower extremity muscle weakness is important from a clinical point of view because it is associated with impaired mobility and reduced quality of life in humans with type 2 [96]. Electrophysiological studies indicate a decrease in the number of estimated motor units in humans with chronic type 1 and type 2 diabetes, as well as motor unit loss associated with weakness in the distal part of limb muscle [90,97,98]. Additionally, re-innervation following axonal loss, seen as the enlargement of motor unit potentials that reflect re-innervation, has also been found [97,99]. Experimental studies in STZ rats show similar results, including denervation and re-innervation at the NMJ, sprouting associated with re-innervation, and an enlargement of motor unit potential by electrophysiological analysis [10,46]. Denervation in the NMJ appears to be particularly strong in the distal part of the limb. MRI studies have indicated that there is a clear distal–proximal gradient of muscular atrophy in humans with diabetes, seen as pronounced loss of lower leg muscle distally and no atrophy proximally [100,101]. This distal–proximal gradient has also been observed electrophysiologically by identifying motor unit loss in the lower leg muscle of humans with diabetes [97]. Ultimately, as autopsy studies show, distal muscles are almost completely denervated and show severe neurogenic atrophy [42]. 

When considering the mechanism of NMJ denervation, it is interesting to note that intranasal delivery of insulin slows the progression of denervation of NMJ more so than its subcutaneous administration [46]. Intranasal delivery targets insulin to the nervous system without significantly altering blood levels of insulin or glucose [102]. Since insulin receptors are expressed both in sensory neurons and MNs, these data suggest that insulin signaling, which has a protective effect on neurons, plays a critical role in preventing the progression of NMJ loss [103,104]. However, since muscle atrophy, likely due to denervation, only occurs in the distal part of the limb, NMJ impairment cannot be explained by insulin signaling abnormalities alone but may be caused by a combination of other well-known DN mechanisms.

Interestingly, muscle weakness is not limited to the foot and ankle areas where denervation of the NMJ and obvious muscle atrophy occur, but also extends to the knee joint, where muscle atrophy does not occur [15,16]. This suggests that a dysfunction of the post-synaptic cell (i.e., muscle fiber) is also a factor in the pathogenesis of muscle weakness. There are already a number of excellent review articles on this issue, so this will only be discussed briefly here [105,106,107,108,109,110]. Muscle weakness in the areas without obvious muscle atrophy is thought to be caused by a combination of DN and disorders that occur in the muscle itself. In these areas, muscle weakness is related to an increase in intramuscular fat or other noncontractile tissue deposits, oxidative stress, and mitochondrial dysfunction, which induce loss of muscle fibers in humans with diabetes [17,111,112,113]. Diabetes also accelerates sarcopenia [110]. At the cellular level, diabetes induces excitation-contraction uncoupling and disruption of Ca^2+^ handling, and these alterations may be related to muscle weakness [114,115]. Type-dependent atrophy of muscle fibers is probably also associated with muscle weakness, but there are conflicting results. For example, while there are reports of selective atrophy of type 2 fibers in animal studies, other reports suggest an increase in type 2 fibers in humans with diabetes [116,117]. 

### 2.4. Differences in Vulnerability to Diabetes between MNs

The susceptibility to effects of diabetes seems to depend not only on the length of the axon, but also on the type of α-MN. Some studies have reported regional differences in diabetic motor neuropathy in experimental models of diabetes. There seems to be a similar difference between α- and γ-MNs, but this will be discussed later; only α-MNs will be described here [48,49]. For example, MNCV has been shown to be further decreased in sciatic/tibial branches to the triceps surae muscles than in those innervating foot muscles in STZ rats [118]. Another report has also indicated that STZ-induced diabetes is associated with differential effects on individual branches of the sciatic nerve [119]. In that study, the MNCV of six branches of the sciatic nerve in 2-month STZ rats was examined and markedly reduced in the nerves to the gastrocnemius, soleus, tibialis anterior, and extensor digitorum longus muscles. However, the branch to the plantaris muscle remained unaffected [119]. Our recent study that used retrograde labeling techniques also indicated that such differences were observed between α-MNs innervating different types of muscle fibers [50]. The number of retrogradely-labeled soleus (slow muscle) α-MNs showed a severe reduction when compared to medial gastrocnemius (fast muscle) α-MNs in a STZ-induced diabetes model [50]. In this study, 3kD dextran was used as a tracer agent. Because labeling with 3kD dextran is predominantly achieved by diffusion into the axonal cytoplasm, this method does not rely on axonal transport machinery that is typically disrupted after induction of diabetes [78,120]. Therefore, decreases in the number of retrograde-labeled MNs may reflect severe axonal damage that extends more proximally than the NMJ. It is unclear what causes the difference in the vulnerability of MNs to diabetes. Previous studies suspect a relationship with different vulnerabilities to hypoxic oxidative stress caused by alteration of nerve blood supply or by mitochondrial dysfunction, since succinate dehydrogenase activity and other factors vary considerably among motor neuron types. Therefore, the effects of ischemia and oxidative stress would be expected to vary considerably, leading to differences in susceptibility to oxidative stress [50,119,121]. Differential diabetic effects have not only been observed in α-MNs but also in muscle fiber type in type 1 diabetic rats. In slow-twitch muscles, contraction and relaxation times are prolonged without apparent morphological alterations. Conversely, contraction times of fast-twitch muscles have been observed to be unaffected; however, a decline in tension and fiber atrophy were observed [50,122]. Thus, it is possible that type-dependent changes in muscle fibers affect MNs retrogradely. However, experiments on humans with diabetes have shown exactly the opposite effect of fast-type motor unit loss in humans with type 2 diabetes [123]. Differences in the vulnerability of MNs to diabetes are important issues when studying the mechanisms of motor neuron disorders and, therefore, an area for future research.

### 2.5. γ-MNs 

Most of the clinical and experimental research into neural circuits that control muscle spindles have concentrated on functional and structural alterations of the primary afferent nerve, while γ-MNs have been overlooked. However, γ-MNs innervate intrafusal muscle fibers of the muscle spindles, and activation of γ-MNs is essential to maintain and modulate the sensitivity of muscle spindles [34,39]. If γ-MNs were injured by DN, it would be expected to reduce the activity of afferent fibers from the muscle spindles, resulting in a diminished stretch reflex, reduced maximal muscle strength due to reduced excitatory effects on the spinal cord via the stretch reflex arch, and reduced motor sensation that may disrupt fine motor control [34,124,125]. However, morphological observation in clinical and experimental studies have only focused on the degeneration of sensory endings of muscle spindles [126,127]. Furthermore, in motor dysfunctions, such as balance and gait deficits, where dysfunction of the stretch reflex may involve muscle spindles or stretch reflex arches, only afferent fiber abnormalities have been of interest [128,129]. Due to these circumstances, only a small amount of information on the effect of diabetes on γ-MNs is available, which is presented below.

Anatomical observations indicate that the intrafusal fibers of humans with diabetes show severe atrophy and the γ-motor nerve is sparse, but there are only a few fine γ-motor axons and their terminations in motor endings were seen in the intrafusal muscle fibers [126]. A study of muscle spindle function in muscles around the ankle joint in humans with DN showed decreased sensitivity of muscle spindles to vibratory stimuli, suggesting the presence of γ-MNs disorders [125]. In an experimental study, we reported the morphological alteration of MNs in STZ-induced diabetic rats using retrograde labeling techniques (3kD dextran was used as a retrograde tracer agent) at 12 and 22 weeks STZ rats, which suggested that γ-MNs are more likely to be affected by diabetes compared to α-MNs [48,49]. In this study, the distribution of average soma diameters in the retrogradely-labeled medial gastrocnemius MNs of control animals was bimodal, with larger groups corresponding to α-MNs and smaller groups, to γ-MNs [130]. Meanwhile, the number of smaller medial gastrocnemius MNs was reduced in 12-week STZ rats. By 22 weeks, diabetic animals had virtually no small medial gastrocnemius MNs and the size distribution became unimodal [48,49]. Accompanying the loss of smaller MNs, muscle spindles showed motor denervation atrophy [49,131]. The mechanism of vulnerability to diabetes in γ-MNs is not clear. However, it is well known that small fibers are more sensitive to hyperglycemia than large fibers, and small-fiber dysfunction is more prevalent than large-fiber dysfunction in DN after a long duration of type 1 diabetes [132]. Such a difference may be related to different vulnerabilities to diabetes probably related to the fact that γ-motor fibers are much smaller than α-motor fibers. Symptoms of γ-MN disorders may be difficult to distinguish from sensory neuropathy because they appear as sensory deficits of reduced muscle spindle sensitivity, which may lead to underestimation of MN disorders caused by diabetes.

## 3. Alteration of Corticomotoneuronal Pathway

### 3.1. Primary Motor Cortex and CST

The motor commands are generated by the interaction of various brain regions and are outputted through the motor cortex, which acts on the brain stem nucleus and spinal MNs to bring about voluntary movement. The former is an indirect pathway to the spinal cord via the nucleus of the brainstem, also called the extrapyramidal tracts, and the latter is a direct pathway that sends motor commands directly to the spinal cord, called the pyramidal tract or CST. Functions of both tracts have been classically divided into two systems: the CST, which controls voluntary movement, and the extrapyramidal system, which controls involuntary movements, such as posture. However, both systems are now thought to be important for the control of voluntary movements [133,134]. In particular, the CST is considered to be one of the essential systems for the execution of voluntary movements [133]. The CST is a phylogenetically new system that appeared first in mammals and developed predominantly in primates, apes, and humans [133].

**Figure 4:**
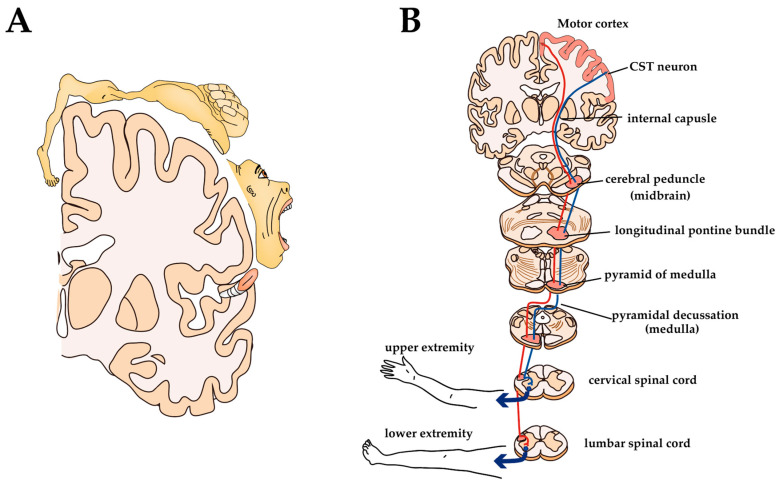
Somatotopic representation of M1 and anatomy of CST in humans: (**A**) Somatotopic motor representations in the human M1; (**B**) Anatomy of CST.

CST originates from CST neurons located in the V layer of certain parts of the cerebral cortex, such as the primary motor cortex (M1), primary somatosensory cortex, and premotor areas [134]. It is classically believed that the M1 of mammals is organized as a ‘homunculus’—a motor representative map of the body in the brain such that contiguous parts of the body are controlled by neighboring cortical regions (Figure 4A). Recent experiments have shown that the fine representations of body regions are overlapping, non-continuous, and flexible, but broadly, it remains the case that there is a body part reproduction in the motor cortex [135,136]. For example, the upper limb (forelimb) region contains CST neurons that project to the cervical spinal cord and the lower limb (hind limb) region contains CST neurons that project to the lumbar and sacral spinal cord. CST axons leaving the motor cortex pass through the internal capsule, and most of the axons cross to the contralateral side at the caudal medulla, called the pyramidal decussation. The location of the CST within the white matter of the spinal cord varies by animal species, with the lateral funiculus in humans, primates, and cats located at the bottom of the dorsal column in rodents (Figure 4B) [31,137]. CST axons terminating in the dorsal horn are indirectly involved in motor control by modulating sensory information during voluntary movement, while CST fibers ending in the ventral horn act on MNs to directly control voluntary movement [138,139]. Most CST axons are polysynaptically connected to MNs via subcortical or spinal inter-neuronal systems [33]. The direct connection between CST and MNs predominantly develops in higher primates, apes, and humans [137]. Experimental studies have shown that, although some recovery of function can be expected later, pyramidotomy results in a sharp decrease in the speed, force, and accuracy of movements of hands and feet (i.e., the most distal parts of extremities) [140,141]. The results of these experiments show how important CST is for the control of voluntary movements.

Little is known regarding mechanisms of DN-caused disruptions within the CNS involved in voluntary movement, that is, the mechanisms of impairment that occur in the motor cortex and CSTs in humans with diabetes and models of diabetes. The mechanisms of CNS damage caused by diabetes have been well studied in hippocampal neurons. Studies have shown that humans with diabetes and animal models of type 1 and 2 diabetes have impairments in cognitive function, synaptic plasticity, synaptogenesis, neurogenesis, and cell death caused by diabetes-related oxidative stress and deficits in insulin signaling [142,143,144,145,146,147,148]. However, data from hippocampal neurons may not be directly applicable to other CNS neurons because hippocampal neurons are specialized neurons, more susceptible to oxidative stress than other CNS neurons; they have a high distribution of insulin receptors and are strongly influenced by insulin signaling [149,150]. However, although it is doubtful that the damage is as severe as that of the hippocampus, the brain, with its high oxygen consumption and lipid-rich content, is highly susceptible to oxidative stress [151]. Experiments have shown that diabetes results in the formation of long-lasting oxidative stress in the brain and decreases the activities of enzymatic antioxidants, such as catalase and glutathione peroxidase [152,153]. Furthermore, alongside increased ROS levels, both nitric oxide levels and mitochondrial nitric oxide synthase expression are increased in mitochondria, whereas glutathione peroxidase activity and manganese superoxide dismutase protein content are reduced. Such oxidative and nitrosative stress might contribute to mitochondrial dysfunction causing direct injury to brain neuronal cells [154]. Additionally, administration of anti-oxidant agents, such as dehydroepiandrosterone, prevents damage to the neocortex [152,153,155]. Considering these results, it is possible that oxidative stress may contribute to neuronal damage in the CNS.

### 3.2. Alterations of M1

Little is known about the effect of diabetes on the function of M1 and motor representations. In a clinical study, transcranial magnetic stimulation of the upper extremity motor area indicated that short-term hyperglycemia did not affect excitability of the motor cortex, but humans with long-term type 1 diabetes showed decreased motor cortical excitability [156,157]. Additionally, resting-state functional MRI (fMRI) indicates abnormal local brain activity synchronization and aberrant functional connectivity of the primary motor cortex in humans with type 2 diabetes [158].

**Figure 5:**
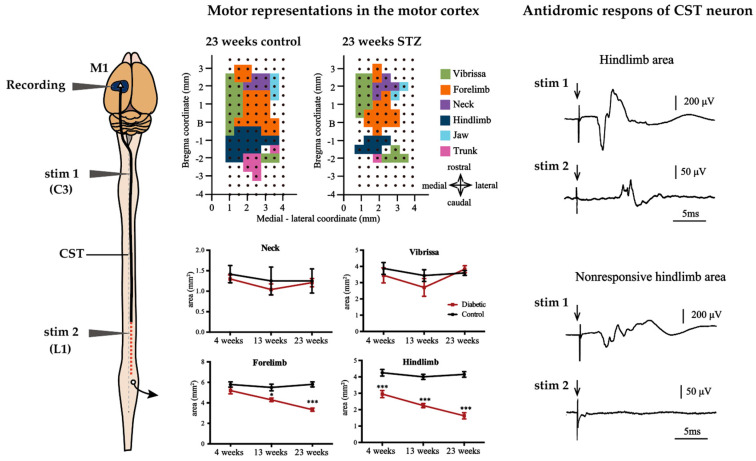
Effect of diabetes on motor representations in the M1 and CST in rats. The area of facial motor areas is not affected by diabetes in M1 of STZ rats, but the areas of forelimb and hindlimb are reduced in STZ rats. In particular, the hindlimb area was severely reduced from the earliest stage of the disease. Length-dependent CST damage may account for this. In 23-week STZ rats, stimulation of CST fibers at the C3 or L1 level, the antidromic field potential of CST neurons can be recorded from the responsive hindlimb area. In contrast, the antidromic field potential of the CST neuron in the non-responsive hindlimb area were recorded when stimulated with C3, but not with L1. This suggests that the CST fibers in the atrophied hindlimb region retain potential excitation up to C3 but lose it at the L1 level. * *p* < 0.05 vs control, *** *p* < 0.001. Arrow indicate stimulus artifact. The figures are modified with permission [29].

An experimental study that focused on alteration of M1 representing the forelimb in a relatively short duration (8 weeks) of STZ-induced diabetes in rats showed a reduction in the forelimb area that was identified by intracortical micro-stimulation. Moreover, these alterations were prevented by insulin treatment [28]. In a recent study, we reported that STZ-treated rats exhibited size reductions in the hindlimb area at 4 weeks and in the trunk and forelimb areas after 13 weeks, with the hindlimb and trunk area reductions being the most severe after 23 weeks [29]. Interestingly, size reduction of the M1 tended to be more severe in areas of the body farther from the cortex (see Figure 5). Conversely, in contrast with clinical studies, cerebral cortex current thresholds for evoked movements were not altered in either of the studies [28,29].

Morphological abnormalities of the M1 and motor-related areas have also been reported. In clinical MRI studies, a widespread reduction in gray matter and white matter volumes and densities were reported. In the corticomotoneuronal pathway, middle-aged humans with type 1 diabetes showed frontal gray matter atrophy, including the M1, independent from cardiovascular risk factors and diabetes complications [159]. Additionally, a decrease in the cortical surface area of the paracentral lobe corresponding to the M1 and primary somatosensory cortex of the lower extremity were reported in humans with type 2 diabetes [160]. The gray matter of M1 and premotor cortex gets thinner in humans with type 2 diabetes [161,162]. Most MRI studies indicate atrophy of motor-related areas; however, they did not describe it in detail because most of these studies focused on the relationship between cognitive function and alteration of gray and white matter volume in humans with diabetes [160,162,163,164]. In an experimental study, 8 -week STZ-induced diabetic rats showed degenerative changes in neurons and glia of the cerebral cortex with perivascular and mitochondrial swelling; however, these alterations predominantly occurred in axons and myelin [165]. Furthermore, dendritic morphological changes, spine density, and length of dendrites of pyramidal cells (probably including CST cells) have been shown to decrease in 16-week STZ rats [166]. Meanwhile, long-term STZ in rats (over 50 weeks with diabetes) induce wide-area brain lesions, including a decrease in the volume of neocortex and the number of neocortical neurons [167,168]. Similar pathological alterations were reported in a study of autopsy of humans who had long-term diabetes [169]. These subjects, however, develop angiopathy [169,170]. Therefore, it was unclear whether pathological changes in the brain were caused by diabetes itself or ischemia. There is little information on models of type 2 diabetes. At least, the number of cortical neurons seemed to be maintained in Goto-Kakizaki rats [171].

### 3.3. Alterations of CST

A decrease in the volume of CST was reported in humans with both type 1 and type 2 diabetes [26,163,164,172,173]. However, there is a report that did not show alteration of the M1 and CST [24,174]. Interestingly, a similar reduction in gray matter and white matter volume and density, including decrease in the CST volume, was observed in not only humans with diabetes but also obesity without diabetes [175]. Electrophysiological studies have indicated that central motor conduction delay is observed in human with both type 1 and type 2 diabetes [176,177,178,179]. However, the reduced conduction velocity of the CST may not have a noticeable effect on the motor function of humans with diabetes. This is because a study examining the involvement of the CNS in postural instability in humans with DN reported that transmission delays occurring in the CNS did not correlate with motor deficits, and the correlation was found only with peripheral neuropathy [180]. A small number of studies have been negative for reduced conduction velocities in the CST, so the reduction in CST conductivity velocities in humans with diabetes remains controversial [157,180]. Although slightly variable, the balance of results from the available literature suggests that the motor cortex and CST are susceptible to the effects of diabetes. It seems a reasonable interpretation that the differences in results are due to differences in duration and severity of diabetes.

In experimental studies, it is known that the conduction velocity of motor descending pathways in both the CST (pyramidal tract) and the rubrospinal tract (extrapyramidal tract) is reduced in long-term STZ-induced diabetic rats [29,181]. Data on conduction velocities in extrapyramidal pathways other than the rubrospinal tract are unknown. Conduction velocities in descending motor pathways were significantly reduced in STZ rats compared to controls, after 6 months of diabetes [29,181]. Interestingly, antidoromic firing of CST cells is observed when the CST fibers originating from the cortical hindlimb area, which are decreased in size, are stimulated in the cervical spinal segment, whereas stimulation in the upper lumbar spinal segment does not evoke CST cell firing. This indicates that the axons of CST cells derived from the motor cortex of the hindlimb remain excitable up to the level of the cervical spinal cord, but lose their excitability in the lumbar spinal cord, where they project to (see Figure 5) [29]. This is consistent with our observation that retrograde-labeled CST neurons projecting to the lumbar segments decreased in number following tracer injection into the spinal cord in diabetic animals, but that CST neurons projecting to cervical segments were preserved [29]. These data suggest that diabetes not only decreases the conduction velocity of the CST, but may also cause severe dysfunction at the distal part of the CST with long axons. Considering the fact that size reduction of hindlimb area induced by only 4 weeks of diabetes because electrophysiological properties of MNs are relatively preserved, it seems that synaptic transmission between CST and MNs were impaired from a very early stage of diabetes [43]. A recent study also reported disorder of synaptic transmission to MNs of the lumbar spinal segment from M1 in STZ rats. In this study, motor neuron potentials were severely attenuated that were recorded from the sciatic nerve evoked by direct current stimulation of M1 in only 2 weeks after STZ injection [182]. Morphological analysis also showed a reduction of synaptic vesicles at synapses in the brains of 8 weeks STZ rats [165]. The impaired synaptic transmission observed from early diabetes resembles the impairments that occur in the NMJ, for example, decreased transmitter rerelease and morphological abnormalities of nerve terminals [92,93,94]. These data suggest that similar axon length-dependent neuropathy may occur within the central nervous system as in the PNS. In PNS, delayed axonal transport of cytoskeletal proteins results in a larger number of cytoskeletal proteins in the proximal region of the axon, which increases in size, whereas fewer cytoskeletal proteins reach the distal axons that show a size decrease is thought to be a contributing factor to length-dependent neuropathy [79,80,81,82,83]. In fact, similar to PNS, cortical neurons of 8 weeks STZ rats show enlargement of the proximal axon and loss of synaptic vesicles in the nerve terminal [165]. Additionally, in the diabetic rat brain, glycation of brain actin and neurofilament phosphorylation were observed, which may be related to reduced slow axonal transport [183,184]. Although no data exist on CST, axonal transport in the CNS may be similarly altered to that in the PNS, as axonal transport in the olfactory nerve, one of the cranial nerves, has been shown to be reduced [87].

### 3.4. Possible Movement Disorders Caused by Diabetes-Induced Lesions of M1 and CST

There is almost no information on the details of how much the size reduction of the motor cortex and CST dysfunction produce motor impairment in humans with diabetes and diabetes models. It is important to note that although it is clinically believed that CST injury causes so-called “pyramidal tract syndrome,” such as motor palsy, in reality, CST injury alone does not cause severe motor palsy, such as stroke hemiplegia. This is because CST injuries observed in clinical practice involve a significant number of other motor descending pathways, and pure CST lesions are unlikely to occur in clinical cases. For example, although a lesion to the posterior limb of the internal capsule is believed to cause “pyramidal tract symptoms,” in reality, CST accounts for only a small percentage of this area, and damage to the posterior limb of the internal capsule is a mix of CST and other motor descending pathways [185]. Experimental studies have shown pure symptoms of lesions in the motor cortex and CST. It is known that if the motor sensory cortex of rhesus monkeys has been removed, voluntary movements have been shown to recover considerably, except for fine movements in the distal upper limb [186]. In monkeys, CST lesions also disrupt interlimb and intralimb coordination during basic locomotion, and cause muscle activation to produce dexterous arm and foot digit movements with decreased speed, force, and accuracy [141,187]. Moreover, cats with experimental CST sections at medullary pyramids are not impaired in simple gait movements, but when they try to walk on ladders or thin bars, they are unable to move and either slip or fall [188]. As described above, symptoms vary slightly from species to species, but pure CST injuries result in a loss of speed, force, and accuracy of movement, mainly in the distal extremities, but not so much that a distinct motor paralysis occurs. Thus, even if motor deficits due to motor cortex or CST lesions do occur, symptoms would be expected to include disorders of fine skill movement and motor coordination and decrease in movement speed and balance. Although most of these symptoms are also observed in real patients, it is difficult to strictly distinguish between the factors that contribute to motor deficits because they also overlap with symptoms of sensory deficits, MNs, and myopathy caused by DN [2,3,4,112]. Experimental studies also indicate that motor dysfunctions in rats with type 1 diabetes were correlated with a significant reduction in presynaptic terminals around the MNs, meaning that changes in spinal neural circuitry that drive motor commands from MNs contribute to the development of motor deficits [73,189]. These data suggest that CNS dysfunction is involved in motor dysfunction, such as lower extremity muscle weakness in humans with diabetes, although there is some debate as to whether this is due to CST disorders. Clarifying the details of motor deficits caused by motor cortex and CST disorders will be a challenge for the future.

### 3.5. Possible Therapies on Corticomotoneuronal Pathway Lesion in Diabetes

Different to the PNS, which has the potential to grow new axons after injury, it is quite difficult to successfully achieve axonal regeneration in the adult CNS. This is due to several environmental mechanisms limiting CNS axonal regeneration. For example, the formation of a glial scar, which predominantly consists of reactive astrocytes and proteoglycans, act as barriers to axons [190]. Additionally, the presence of myelin-derived axon growth inhibitors also disturbs the regeneration of the CST [191]. Thus, the regeneration of CNS neurons is quite limited, but among them, CST neurons are known to be the least capable of generating axonal regeneration beyond the site of injury. For example, implantation of a peripheral nerve tissue or a Schwann cell-seeded guidance channel into the transected spinal cord to eliminate extracellular matrix molecules or myelin-associated inhibitors allow regeneration of the bulbospinal tract, such as the reticulospinal tract, while little or no regeneration of the CST occurs [192,193,194,195]. Nevertheless, functional recovery occurs after CST injury because CST neurons generate alternative descending pathways that bypass the lesion site and deliver motor commands to the spinal cord [196]. For example, monkeys that underwent unilateral lesions of the medullary CST showed an initial contralateral flaccid paralysis, after which motor function rapidly recovered. Electrophysiological examination of the neural circuitry after recovery revealed the formation of an alternative descending pathway through the reticulospinal tract [197]. In addition, in rats with disrupted unilateral cerebral cortex, CST fibers originating from the intact cerebral cortex sprout and form new synapses with reticulospinal neurons to form an alternative descending pathway also through the reticulospinal tract [198]. The formation of these alternative pathways appears to occur in various regions of the CNS. The use of alternative descending pathways to restore motor function has also been observed in the spinal cord. If the CSTs are lesioned, the remaining CSTs will strengthen their connection to propriospinal neurons or form new synapses to transmit motor commands to MNs via the propriospinal tract [199,200]. Recent studies have reported the formation of alternative pathways from the premotor cortex through the deep cerebellar nuclei to the spinal cord after lesion to the primary motor cortex [201]. The same is true for M1 injuries. Both clinical and experimental studies have shown that successful M1 lesion recovery hinges on the brain’s ability to re-map motor functions to intact, functionally allied brain regions [202,203,204]. Therefore, it is strategically important that the recovery of function from M1 and CST injuries does not promote the regeneration of damaged neural circuits, but rather promotes the formation of neural circuits that compensate for the lesioned function. However, there is evidence that this re-map may be inhibited in 4-week STZ mice that did not improve after insulin administration [12]. These data show that restoring M1 and CST functions is not only about treating diabetes; it is necessary to create intrinsic and extrinsic factors that accelerate plastic changes in the CNS.

Rehabilitation is very important to accelerate motor function recovery after M1 and CST lesions. In monkeys, after M1 lesion recovery, motor behavior can be promoted by intensive use of the affected limb during postlesion training [205,206,207]. In rats with a lesioned CST at the spinal cord, rehabilitation increases collateral sprouting of lesioned CST fibers rostral to the injury and increase cortical levels of growth associated protein-43 [208]. It is also known that sprouting of CST nerves and the formation of alternative pathways that mediate recovery of motor functions are accelerated by electrical stimulation of the motor cortex [209,210,211]. Conversely, disuse of an impaired forelimb by disruption of one side of the M1 interferes with its functional recovery [207]. This means that the formation of alternative pathways for CST recovery is likely to be accelerated in an activity-dependent manner in the brain. A recent clinical study showed that only 2 weeks of toe resistance training improved pinch force and knee extension force in humans with DN [212,213]. Gains in strength during such short-term training have been attributed to adaptations within the CNS [214,215]. Thus, data indicate that exercise therapy has the potential to promote activation of the cortico-muscular pathway in humans with diabetes. It is also important to create an environment that elicits the effects of rehabilitation on the recovery of brain functions. It has recently been shown that administration of edonerpic maleate (a Collapsin-response-mediator-protein 2 binding compound that is a downstream molecule of semaphorin and is thought to be related to synaptic plasticity and learning) alone does not restore motor function after motor cortex injury. However, combined with rehabilitation, edonerpic maleate has been shown to accelerate motor function recovery compared to rehabilitation alone [216]. Combining rehabilitation with the administration of molecules that aid neuroplastic changes may be the key to improving CNS damage in diabetes. However, in terms of surrounding environment, many of the brain environmental conditions caused by diabetes are thought to prevent the brain’s plasticity from recovering functions [217]. For example, astrocytes are critical for normal CNS function and play an important role in neuronal plasticity [218,219]. However, the cerebellum of 12-week STZ rats shows glial activation, cell death, and loss of glutamate transporters in astrocytes [220]. Not only do these changes reduce the support of astrocytes to brain plasticity, but also mean exposure to excitotoxicity due to reduced glutamate transporters and disruption of nutrient supply to neurons. These may turn the astrocytes into a barrier to the recovery of brain function [220,221]. Conversely, reactive astrocytosis has been observed in the cerebral cortex, hippocampus, and cerebellum of STZ rats, which has been reversed by the administration of vitamin E, an anti-oxidant, and melatonin, a direct radical scavenger [222,223]. While astrocytes are only one example, it seems important to treat M1 and CST diabetes-caused injuries to not only provide an environment conducive to rehabilitation and functional recovery, but also to identify and treat elements that may interfere with the plastic changes in neural circuits caused by diabetes. However, few studies have been conducted in this regard, and further research is needed.

## 4. Conclusions

As discussed in this review, there appears to be no doubt that diabetes-related disorders of the cortico-muscular pathway exist. These CNS disorders that occur could add a new explanation for movement disorders in humans with diabetes. However, data gaps remain, including the molecular mechanisms, effects on voluntary motor control, and potential treatments. In particular, the molecular mechanisms of these disorders remain unexplored, and further research is warranted.

## Figures and Tables

**Table 1:** Characteristics of the animal models of diabetes that appear in this review [35].

Model	Induction Mechanism	Type of Diabetes
STZ rats	Chemical induction	Type 1 diabetes
Bio-Breeding (BB) rats	Spontaneous	Type 1 diabetes
Zucker rats	Spontaneous	Obesity model of type 2 diabetes
Goto–Kakizaki rats	Spontaneous	Lean model of type 2 diabetes

## Abbreviations

DN	diabetic neuropathy
PNS	peripheral nervous system
DRG	dorsal root ganglion
BBB	blood-brain barrier
STZ	streptozotocin
BB	Bio-Breeding
MNCV	motor nerve conduction velocity
NMJ	neuromuscular junction
M1	primary motor cortex
CST	corticospinal tract
MN	motoneuron
ACh	acetylcholine
ROS	reactive oxygen species
AGEs	advanced glycation end-products
RAGE	receptor for AGEs
